# Conceptualization of head-heart-hands model for developing an effective 21st century teacher

**DOI:** 10.3389/fpsyg.2022.968723

**Published:** 2022-10-14

**Authors:** Md. Aminul Islam, Seriaznita Binti Haji Mat Said, Jameela Hanoon Umarlebbe, Farid Ahammad Sobhani, Sadia Afrin

**Affiliations:** ^1^Faculty of Business and Communication, Universiti Malaysia Perlis, Arau, Malaysia; ^2^Faculty of Business and Entrepreneurship, Daffodil International University, Dhaka, Bangladesh; ^3^Faculty of Social Sciences and Humanities, University of Technology Malaysia, Johor Bahru, Johor, Malaysia; ^4^School of Business and Economics, United International University, Dhaka, Bangladesh

**Keywords:** teaching, Bloom's Taxonomy, 3H approach, effective teacher, classroom management

## Abstract

Celebrities, athletes and even politicians are frequently identified as role models for most impressionable students. But the reality is, outside of their own home, one of the greatest role models in a student's life is standing in front of the classroom. Teachers are people to look up to and emulate. This paper attempts to discover some of the essential qualities and skills that would help teachers and the students they teach achieve their full potentials, and also some key characteristics that contribute to teachers and instruction effectiveness. This paper also attempts to highlight the features of 3H (Head, Heart and Hands) model in effective instructional approach and theorizes that the 3H attributes in combination with Bloom's Taxonomy will produce effective 21st century teachers. Hence, this article endeavors to create awareness and contribute to the transformation of teachers toward reflective and visionary; confident and competent teachers. After all, effective teachers will inspire their talented students to live up to their full potentials. Effective 21st century teachers are in the best position to shape and equip students with the desired 21st century skills.

## Introduction

At the entrance gate of a university in South Africa the following message was posted for contemplation:

“*Destroying any nation does not require the use of atomic bombs or the use of long-range missiles. It only requires lowering the quality of education and allowing cheating in the examinations by the students.”*

21st century students are affected by the COVID-19 pandemic that is still ravaging the world while the Industrial Revolution 4.0 impacting the globe. The policymakers, educators and scholars, on the other hand, are grappling with the answers to the question of how education should address these present challenges. Lim and Grant (2014) proposed closing Asia's enormous gaps in basic learning by equipping students with 21st century skills, such as inter- and multidisciplinary thinking, creativity, critical thinking, resilience and cross-cultural competencies. By the same token, the aims of education for sustainable development (ESD) are to help people to develop the attitudes, skills, and knowledge to make informed decisions for the benefit of themselves and others, now and in the future, and to act upon these decisions (UNESCO, 2012). Shaaruddin and Mohamad (2017) in their research on identifying effectiveness of active learning strategies also concluded that positive learning environment, direct interaction between lecturers and students, communication skills, respect of others' opinions, allow students to get engaged with the learning activities and encourage participation. In addition to improving test scores, effective teachers are also required to manage classroom behavior, give accurate instruction, foster critical thinking, and create emotionally safe spaces for students to grow socially and emotionally (Pianta and Hamre, 2009; Cohen, 2011).

With the advancement of new technologies, the kind of innovation that the world really needs for educational renewal in these challenging times lies in nurturing and strengthening the human factor. This human factor in question is an effective teacher who knows what is beneficial for students' holistic education. Aligned with the purpose of this paper, a teacher is construed as an instructor who teaches tertiary level students, and the students comprise of young adults who enroll in college or university courses. An effective teacher is envisioned to prepare college or university students with useful skills, and to improve their knowledge and competencies in their desired fields. Therefore, education today calls for teachers to be highly skilled knowledge workers who constantly improve both their personal and professional achievements. Not forgetting that innovation is crucial for creating new sources of growth through increased productivity and efficiency, teachers are also required to be agents of innovation (OECD, 2010).

This is very much true in the field of education. The incorporation of innovation into curricula and instructional strategies can enhance student learning and better prepare them for the continuously evolving demands of the 21st-century labor market (OECD, 2009). However, in a fast-changing world, delivering more of the same knowledge will not be enough to meet the challenges of the future. Routine cognitive skills, which are the ones that are easiest to teach and measure, are also the ones that are easiest to digitalize, automate, and outsource, which is perhaps the most difficult conundrum facing teachers today (Schleicher, 2012). Previously, instructors could assume that the lessons they imparted would stick with their students for the rest of their lives. Given that students can easily access content *via* Google, that routine cognitive tasks are being automated or outsourced, and that the nature of work is rapidly changing, inasmuch as education systems must put a much greater emphasis on preparing students to be lifelong learners and to manage the complex ways of thinking and working that exist today. Students, equally, must be capable of positioning themselves and repositioning themselves in a world that is rapidly changing, as well as constantly adapting and learning.

Although teachers nowadays are usually high achievers academically, there is a need to provide adequate training to prepare them to be capable and “fit to teach” the 21st century students. The adoption and practices of appropriate learning approaches in designing and delivering contents of courses offered is crucial in producing an effective teacher who complies with the standards universally accepted by the society. The “head, heart and hand” is a holistic approach, first put forth by Orr in 1992 and expanded by Sipos et al. (2008). The paradigm illustrates the all-encompassing nature of transformative experience and ties relational knowing and involvement to the cognitive (Head) and affective (Heart) domains. Additionally, it connects critical reflection with the psychomotor (Hands) and emotional (Heart) domains. Pugh (2002) pragmatic construct of transformational learning experience offers an analytical tool for assessing transformative experiences through increased perception (cognitive), expanded value (affective), and active application of learned concepts (psycho-motor). In line with the purpose of this paper, these standards are attributable to three domains of learning: Cognitive/Knowing (Head), Affective/Feeling (Heart) and Psychomotor/Doing (Hands) or henceforth known as 3H Model.

The 3H model proposes that any effective teaching or learning activities should embed the Head as essentially responsible for imparting knowledge, Heart that inculcates in an individual the values and a sense of appreciation, and Hands component that encourages active involvement during teaching and learning activities. The Heads-on creates the knowledge culture, Heart-on influences reflection and Hands-on helps develop student's thinking and living skills. Tan et al. (2021) in their article on 3H framework in curriculum review found that the Head, Heart and Hands framework and its self-similarity property provide a potential basis for a holistic approach to curriculum review. Ellis (2004), cited the Collins (1995) that students ought to have minds-on and/or heads-on experiences during hands-on activities. It is also concluded that while hands-on, the students are learning by doing but while minds-on learning, the students are thinking about what they are doing and learning.

## Background of study

This paper staunchly argues that the role of a teacher in education is highly significant. The learning process becomes effective when the teacher has successfully enabled the learners to acquire necessary knowledge and skills. There are noteworthy differences of achievements of educational goals of institutions despite having similar inputs and academic achievement of graduates. The inputs of a good quality education should be coupled with well trained teachers, high quality students and a balanced curriculum (Babalola, 2004). Thus, teachers play the most important role in organizing and delivering lessons that contribute to skilled and knowledgeable students. Balci (2013) noted that teachers are often given opportunity to attend in-service training, seminars, webinars, and short courses to improve themselves. Consequently, teachers are expected to maximize their performance and contribute positively to producing quality students. This is supported by the findings of a research that showed a significant relationship between the teachers' efficacy beliefs and teachers' “motivation and persistence over the course of a career”(Tschannen-Moran and Hoy, 2001, p. 803).

Education in the 21st century has specific demands that expect students' learning be consolidated with new technologies. Apropos of that, it clearly indicates that teacher instructional quality is anticipated to parallel the 21st century curriculum goals which are to equip students with useful skills and to improve their knowledge and capabilities in their chosen fields. Therefore, the gap that this paper endeavors to straddle is despite the overwhelming professional demands, teachers could still be guided to employ the 3H aspects to live up to the challenges faced by the 21st century education. The policy focus has shifted from looking up to the bureaucracy to looking outwards to the teachers and subsequently the school, from the provision of education to outcomes (Schleicher, 2012). The issue now is to encourage user-generated knowledge among teachers on the front lines. The past was about the delivery of knowledge. Hence, teachers in the past were frequently left in isolation in the classrooms with extensive directives over what to teach. The most cutting-edge educational systems now have high expectations of the students and a clear understanding of what they should be able to perform. The systems then prepare the teachers pedagogically and methodologically and give them the resources needed to ensure the curriculum goals are achieved and the students' needs fulfilled.

In addition, today's teachers are encouraged to embrace diversity through varied pedagogical techniques, unlike in the past whereby students of various capabilities were taught in comparable ways. Significantly these developments have impacted the leadership of schools and educational systems as well as instructors, students, and teaching and learning processes. The past emphasized curriculum-centered whereas the present is learner-centered, and thus, the goal of the past curriculum was standardization and conformity; while curriculum today is about being inventive and personalizing educational experiences. Teachers are being asked to personalize learning experiences to ensure that every student has a chance to succeed in dealing with the growing cultural diversity and differences in learning styles in their classrooms. From the pedagogical perspective, this means teachers must design learning in ways that allow individual students to learn in the ways parallelizing their progress.

According to Gazibara (2013), the combination of 3H to transform the learning culture that emphasizes the entirety and interactivity of a human being has been propagated by researchers such as Stoll and Fink (1998), Orellana (1999), and Bruner and Porath (2000) since the onset of 21st century. One of the instructional approaches that embody 3H concepts and meet the challenges of the 21st century education is Student-Centered Learning (SCL). According to Collins and O'Brien (2011) SCL is a method of teaching whereby the students are in control of the subjects, resources, activities, and progress of learning. SCL puts students as the center of the teaching-learning process. The teachers encourage students to learn independently and collaboratively while providing them with the skills needed to do so. McCombs and Whisler, 1997 state that students are viewed as active participants in the learning process who have unique perspectives and needs that should be taken into account. In addition to that, the goal of student-centered learning environments is to build on existing knowledge since prior knowledge strongly affects future learning (Froyd and Simpson, 2008). Therefore, if SCL is accomplished properly, it can result in improved learning motivation, knowledge retention, comprehension, and constructive attitudes about the subject material.

This paper problematizes that the constraints which occur in general tertiary educational contexts could be overcome by providing teachers simple guidelines to be an effective teacher who can achieve the highest standard of integrity and professionalism. Hence this paper discusses how teachers could nurture 3H qualities in their students to ensure the role of teacher remains relevant in the ever challenging and demanding 21st century. Against this background, the objective of this paper is to discuss the attributes of 3H in making an effective teacher. Likewise, it examines the following research question: How could the framework of 3H support the development of effective teachers?

## Importance of the study

To date, the worldviews of education continue to evolve with time. Accordingly, teaching in the 21st century requires a paradigm shift. Lecturing alone is not sufficient when humanity is striving to find the best pedagogical techniques and ways of equipping students with balanced rudiments of knowledge, skills, morality, and freedom in education. Although many research have debated that equipment, materials and tools in teaching are important to facilitate learning and enhance student achievement, the core or spark plug of the curriculum is without a doubt a soul that we call “teacher,” which is what this paper will discuss in breadth; how 3H can facilitate teachers to be effective and attain the curriculum goals set by policy makers.

## What is education?

Under this subheading, education is discussed chronologically with relevance to a significant teacher role. As far back as 384-322 BC, Aristotle, the renowned Greek philosopher and scientist stressed the importance of education by noting the following:

“*It is right that we ask [people] to accept each of the things which are said in the same way: for it is the mark of an educated person to search for the same kind of clarity in each topic to the extent that the nature of the matter accepts it. For it is similar to expect a mathematician to speak persuasively or for an orator to furnish clear proofs!*

*Each person judges well what they know and is thus a good critic of those things. For each thing in specific, someone must be educated [to be a critic]; to [be a critic in general] one must be educated about everything.”*

(Aristotle, *Nicomachean Ethics*, 1 1094a24-1095a cited in Sententiae antiquae)

Albert Einstein, 1879–1955, a great physicist took the idea further by postulating that education is what remains after one has forgotten what one has learned in school. What is perhaps a poignant statement made by the American philosopher, Allan Boom, 1930–1992, really captures the transformation of a learned person that his or her world goes from shrouded in obliviousness to lively with prospect. Bloom (1956) claimed that “Education is the movement from darkness to light.” Equivalently, Jean Piaget, a world prominent Swiss developmental psychologist and philosopher in the era of 1896–1980, stressed that “*The principal goal of education is to create men who are capable of doing new things, not simply of repeating what other generations have done—men who are creative, inventive, and discoverers. The second goal of education is to form minds which can be critical, can verify, and not accept everything they are offered.”* (Piaget, 1953).

Reviewing all the quotes from these great minds, one can make a valid assumption that education entails a logical process that facilitates learning. Through storytelling, discussion, teaching, training, or research; knowledge, skills, values, beliefs, and habits of a great teacher are transferred to other individuals. Funneling this idea, an individual would have acquired education after studying particular subject matters or experiencing life lessons that contribute to an insight of something. Most importantly, this process requires instruction of some sort from an individual or composed literature. For this reason, Ezeji (1993) highlighted that teachers' utterances, actions, leadership styles, knowledge of the subject and skills in teaching are still being considered important factors in student learning.

Revisiting the message cryptically engraved on the entrance gate of a university in South Africa, imagine the outcome if students cheat during the examinations and the teacher turns a blind eye to it? The possibilities are that in the future patients may die at the hands of dishonest doctors, buildings may collapse at the hands of unrepentant engineers, money may be lost at the hands of fraudulent economists and accountants, humanity may wither at the hands of unscrupulous religious scholars, and justice may not be honored at the hands of crooked judges and so forth. Consequentially, the collapse of education signals the collapse of the nation. Therefore, Özgenel and Mert (2019) in their study on the role of teacher performance in school effectiveness recommended policy makers and school leaders to establish an effective performance evaluation system to determine teachers' performance to motivate teachers for improved performance.

Obviously, almost all arguments indicate that education encompasses the process of facilitating learning which requires the instruction of some sort from an individual or composed literature. This brings us to the next topic of discussion on this pivotal figure in education - teachers.

## How to nurture traits of an effective teacher?

A study on Swiss primary school teachers' perceptions of their competence in dealing with job challenges revealed that teachers are expected to meet professional requirements concerning: (1) their role as a teacher, (2) capacity to teach and meet students' specific learning needs, (3) managing the classroom effectively, and (4) cooperating with other professionals within the school (Keller-Schneider et al., 2020). Aligned with the purpose of this paper, the discussion gravitates to the first three concerns. These challenges are obviously burdening teachers and over time, without profound ways to support teachers, the teaching profession will not be a popular choice for future generations to come. Graham and Flamini (2021) conducted a study on teacher's quality and students' post-secondary outcomes and concluded that students attending schools with highly experienced teachers with good academic credentials are more likely to have positive post-secondary outcomes. It is interpreted that there is a high probability that the former students could have furthered their education or have obtained secure employment.

How can the teaching profession be reinstated as a respectable vocation? It has to evolve by the adopting transdisciplinary approach. The business model of excellent customer service quality is a plausible solution for a teacher to effectively meet students' specific learning needs. Adapted from Riaz (2016), this model encourages a teacher to treat the students as customers. Mentally, the teacher has to create values for his or her customers through quality products and excellent services by forming the “customer first” mindset, striving to fulfill customers' expectations, being proactive, providing speedy responses and recovery, and delivering value-added services. The teaching profession should also prioritize ethics which ensure highest standard of integrity and professionalism. Teachers' primary duties to their students are outlined in a professional code of ethics, which also describes their place in the lives of their charges. Above all, instructors should conduct themselves with honesty, fairness, and morality in the classroom as well as in interactions with parents and co-workers.

The strategies mentioned earlier should be consolidated with some if not all of the characteristics of a great teacher as espoused by Orlando (2013). According to Orlando (2013), a great teacher values each student's ideas and opinions. Consequently, students will learn to respect and listen to each other, and feel safe to express their feeling. Secondly, a great teacher should be ambitious and set high expectations as generally students will give as much or as little as is expected of them. Such a teacher will also constantly renew the quest of acquiring new knowledge, new pedagogies and incorporate them in teaching. An effective teacher should be able to “shift gear” upon determining that the current lesson plan is not working. Thus, an effective teacher should be flexible and should continuously assess the teaching throughout the lessons and find innovative ways to present materials that is understood by all students. An effective teacher should exhibit professionalism in all areas – from maintaining a positive personal appearance to displaying readiness for lessons, excellent delivery and organizational skills. Orlando (2013) also suggests that an effective teacher should be caring, warm and accessible. This will motivate students to express their feelings and ideas without fear or repercussions. An effective teacher should exhibit sense of belongingness and commitment so that the classroom environment becomes supportive and collaborative. This paper would like to add to this list the up-skilling and re-skilling efforts should be a part and parcel of a teacher's life. This will transform an effective teacher into a skilled leader in the classroom.

Regardless of a great content knowledge of the subject, according to Rubio (2009), one significant element that an effective teacher must possess is classroom management skill. On this matter, this paper adopts the stance that it can be achieved if a teacher is able to influence and motivate students. Every teacher generally finds that it is not an easy task to motivate students. Nonetheless, it is one of the most important responsibilities that a teacher has to carry because unmotivated students will not learn well. Students who are not keen on learning will refuse to participate in learning activities, will not retain information, and some may even show disorderly conduct. A student's motivation may be waning for a variety of reasons: the subject fails to capture the student's interest; the teacher's sterile and uncreative methods bore the student or external factors are distracting the student. More than often, it may even come to light that an unenthusiastic student actually has difficulty learning and is in need of special attention. Boysen (2018) discovered that students cheated more frequently in settings lacking instructor supervision, order, and student involvement. Greater systemic problems are associated with student cheating habits, and students cheat more when their teachers are unaware of them. Therefore, it may be concluded that teachers' classroom demeanor and students' cheating behavior are related. Boysen (2018) had come to a conclusion that teachers can lower the rates of cheating in their classrooms by enhancing the learning environment, particularly in the areas of structure, organization, and student participation, as well as by using more real evaluations that are based on the standards i.e., pop quizzes, presentations, group discussion and other continuous assessments.

**Figure 1:**
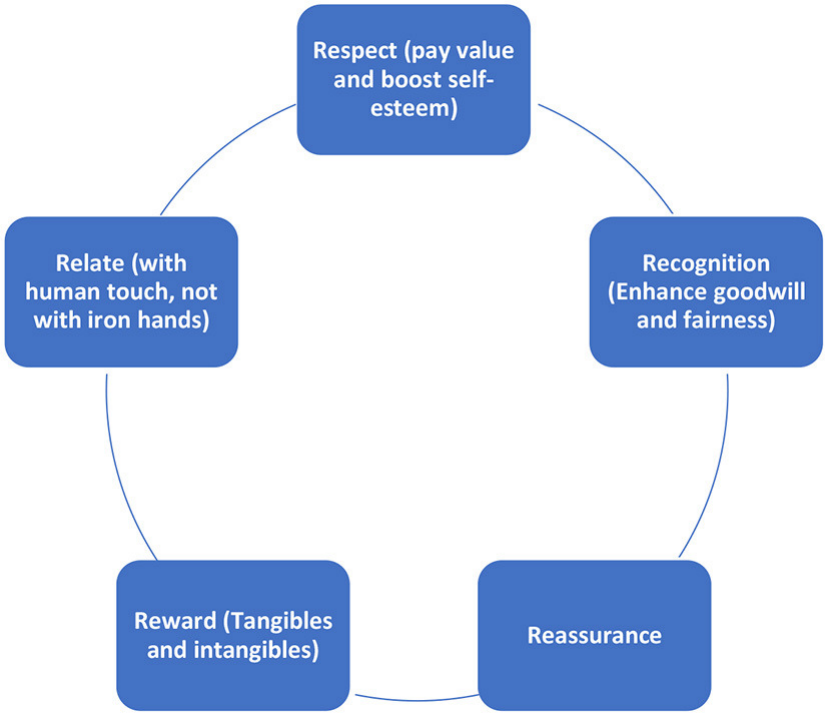
The 5Rs to influence and motivate students for results results. Drawn using source data from Hoban and Nielsen (2010).

Collaborative Problem Solving (CPS) skills which aid both the division of labor and the integration of information from various sources of knowledge, perspectives and experiences are in resonance with Boysen's (2018) argument. CPS framework is generated by the Organization for Economic Co-operation and Development (OECD) and CPS skills are conceptualized to help hone effective teamwork (OECD, 2013). The following diagram (Figure 1) is derived from extant literature on how CPS enhance the creativity and quality of solutions that a teacher can easily adopt in their pedagogy (Hoban and Nielsen, 2010):

It is, thus, obvious that the study of classroom management has become an important aspect of developing an effective teacher. This external support is frequently given by teachers, who are in charge of fostering an environment that encourages and improves students' learning (Johnson, 2017). The support that teachers provide for the growth of students' autonomy, relevance, relatedness, competence, teachers' interests, and teachers' self-efficacy about teaching their subject is how students perceive the teachers' involvement in enabling students' motivation. Regardless of whether students are intrinsically or extrinsically motivated to learn, the teacher's support of their education and creation of the ideal environment will increase this drive.

The last review is allocated to common malpractices among teachers, 3H and how an effective teacher is cognizant of these attributes and employ them in daily lessons.

## Common malpractices among teachers

Teachers are essential to accomplishing learning objectives and play a vital role in students' performance. Their presence in the classroom is regarded as a fundamental “pre-requisite” for learning to occur, for achieving national and regional educational objectives. Lost instructional time can have a negative impact on student learning, overall academic success, and learners' possibilities in life. This section focuses on common malpractices among teachers.

The most common malpractice can be described as unpunctuality. Generally, punctuality refers to arriving or taking place at an arranged time. It takes ample time to complete the syllabus before the end of semester as well as to mark examination scripts and review students' portfolios. All the work requires time. There are times even teachers may have to cancel classes due to personal reasons. In some instances, teachers sometimes come to the class late or finish the class early without fulfilling the learning objectives of the lesson. On the other hand, habitual unpunctuality can create knowledge gaps among the students. Therefore, punctuality does not apply solely to the students, teachers are expected to arrive on time as well. McEwin (1978) remarked that there are teachers who are not punctual and do not seem to leave for work any sooner in the morning. McEwin (1978) also observed that in spite of all the various school regulations and the admonitions in textbooks, without punctual teachers to guide them, children are unlikely to make punctuality a habit.

Absenteeism is another common malpractice documented by previous researches. Absenteeism can be defined as the failure of an employee to attend the scheduled working hours imposed by the workplace or institution. Mfaume and Bilinga (2017) indicated that absenteeism is the most serious type of professional misconduct committed by teachers. As per Finlayson (2009), the performance of the students may be adversely affected because of teachers' absenteeism. Finlayson (2009) in a study conducted on a developing country also confirmed that the more frequently a teacher misses scheduled classes, the worse the student will perform in standardized tests. Moreover, teacher absenteeism correlates with student absenteeism which in turn affects the students' achievements as well as has a negative impact on the education system of a country (Niamatullah et al., 2020).

Several researchers (Dang, 2007; Silova, 2010; Bray and Lykins, 2012; Lao, 2014) have identified private tutoring as the other notable malpractice. It is not a new phenomenon rather it has been practiced for a long time in both developed and developing countries. In many countries, private tutoring has become a lucrative business. Private tutoring is becoming a common phenomenon in Malaysia (Kenayathulla and Ubbudari, 2017). Unlike other countries in the world where private tutoring is banned, in Malaysia the government has legally allowed the teachers to tutor. Sambo (2001) confirmed in a study that classroom teachers in most institutions purposely delayed fulfilling the curriculum objectives so that the students will have to attend their private tutoring sessions. Bray (2003) also identified this as a problem because indigent students cannot afford extra tuitions which hinders their legal rights to quality education. Moreover, since the United Nations Declaration on Human Rights emphasizes equity and equal access to education for all children, ignoring other pupils' opportunities to learn is deemed a significant offense.

Sylvester (2011) in his research on malpractices of teachers concluded the abusive behavior is common in many countries. Teachers play a crucial role not only as an educator but they also play a primary socializing role to help students accomplish fundamental socioemotional requirements like a sense of belonging and esteem. Consequently, repeated verbal abuse by a teacher might damage the potential of a student as it interferes with the student's ability to meet the basic socioemotional needs. It can also harm student's ability of healthy growth. Sometimes teachers intentionally or unintentionally engaged themselves in bullying. It can be through sarcasm, cryptic naming, rejecting late or misidentified work, and embarrassing students who they believe may have behavioral issues in the classroom (Sylvester, 2011). It can significantly harm the mental health of the students. In addition, when a teacher bullies a student, the bullied student may feel unable to take legal action. Rahimi and Hosseini (2015) also discovered that students evaluated teachers who employed punishing tactics as being less effective in their instruction than those who regularly used involvement and recognition strategies. It was also found that students struggled to learn in classes when teachers used punitive measures to deal with disruptive behaviors since these measures were attributed to a decline in students' motivation.

In addition to the factors listed above, the extant literature highlighted other teacher malpractices that demoralize students include lack of caring, sharing, fairness in the classroom, consistency in teaching, helping nature toward students etc.

## Utilizing head, heart and hand concept in teaching

3H as a holistic teaching approach embraces the affective, psychomotor and cognitive domains in equal degree. This paper theorizes that 3H will contribute to a great tidal wave on the ways 21st education should be administered and supported to promote learning among students. This comprehensive framework from the individual's head, heart, and hands is a place to start when modeling changes in the strategy for educational reform that gives the learner a sense of meaning and purpose. Orr (1992) postulated that education should involve application and the disposition of how to generate meaning and value in addition to content or formal knowledge. According to the framework developed by Sipos et al. (2008), “head” refers to using the cognitive domain by engaging in academic study, research, and comprehension of ecological and sustainability principles. In the context of learning practical skill development and physical activity like constructing, planting, and painting, the term “hands” refers to the psychomotor domain in action. Heart is the ability of the emotive domain to establish beliefs and ideals that are then translated into actions (Sipos et al., 2008).

The culture of 3H teaching should adopt Bloom's Taxonomy (Bloom et al., 1956). This paper argues that Bloom's Taxonomy has successfully compartmentalized the essence of 3H in teaching outputs into levels of complexity and specificity. Bloom's Taxonomy also advocates holistically oriented pedagogy which acknowledges the role of a teacher in promoting a balanced growth in the student. This is pertinent in dealing with the 21st century education paradigm, which sees the incorporation of latest technology trends in classroom instruction.

Examining the extensive literature reveals that holistic learning is oriented toward satisfying students Head-Heart-Hand (3H) as synthesized by the keywords that represent the levels of Cognitive (Head), Psychomotor (Hand) and Affective (Heart) domains in Bloom's Taxonomy. As illustrated by Table 1, the skills and keywords are arranged in ascending manner to show the levels of difficulty which an effective teacher should consider in planning classroom instruction and evaluation in each of the three domains.

**Table 1:** Bloom's Taxonomy (Bloom et al., 1956; Simpson, 1972; Abalkheel, 2022).

**Cognitive domain (HEAD)**		
**C1**	Knowledge	Arrange, define, duplicate, label, list, memorize, name, order, recognize, relate, recall, repeat or/and reproduce state
**C2**	Comprehension	Classify, describe, discuss, explain, express, identify, indicate, locate, recognize, report, restate, review, select or/and translate
**C3**	Application	Apply, choose, demonstrate, dramatize, employ, illustrate, interpret, operate, practice, schedule. Sketch, solve, use or/and write
**C4**	Analysis	Analyze, calculate, categorize, compare, contrast, criticize, differentiate, discriminate, distinguish, examine, experiment, question, or/and test, derive
**C5**	Synthesis	Arrange, assemble, collect, compose, construct, create, design, develop, formulate, manage, organize, plan, prepare, propose, set up or/and write
**C6**	Evaluation	Appraise, argue, assess, attach, choose compare, defend estimate, judge, predict, rate, core, select, support, value or/and evaluate
**Affective domain (HEART)**		
**A1**	Receiving Phenomena	Describe, follow., name, select, reply, use
**A2**	Responding to Phenomena	Read, answer, discuss, perform, practice, recite
**A3**	Valuing	Justify, differentiate, study, explain, demonstrate, initiate
**A4**	Organizing Values	Organize, identify, formulate, integrate, arrange, synthesize
**A5**	Internalizing Values	Solve, modify, discriminate, practice, propose
**Psychomotor domain (HAND)**		
**P1**	Perception	Detect, describe, differentiate, isolate, distinguish, choose, select, relate, identify
**P2**	Set	Begin, proceed, explain, move, react, state, show, display, volunteering
**P3**	Guided response	Copy, trace, react, respond, reproduce, follow
**P4**	Mechanism	Assemble, measure, mix, calibrate, dismantle, display, construct, grind, manipulate, mend, fix, heat, sketch, organize
**P5**	Complex overt response	Assemble, calibrate, construct, build, display, dismantle, mend, fix, measure, manipulate, sketch, mix, organize
**P6**	Adaptation	Alter, adapt, vary, change, rearrange, reorganize, revise
**P7**	Origination	Arrange, originate, create, build, construct, design, compose, combine, initiate

Examining the extensive literature reveals that holistic learning is oriented toward satisfying students' 3H as synthesized by the keywords that represent the levels of Cognitive, Psychomotor and Affective domains in Bloom's Taxonomy (Simpson, 1972; Gazibara, 2013; Singleton, 2015). The Head is akin to cognitive and Heart concept is akin to affective domain (Krathwohl et al., 1973).

## How to get started?

Anyone who aspires to be a teacher should ask themselves, what type of teacher best describes themselves? The following is a list of types of teachers derived from the authors' personal encounters and these teachers had made an indelible impression because of their personal eccentricities, number of assignments they gave, pitch of voice they adopted when teaching and non-verbal communication they displayed: The teacher is the coach who teaches students valuable lessons in life in addition to resolving students' academic issues. The rowdy teacher is the one who always gets on students' nerves. The teacher is strict with students, and the many punishments keep the students on their toes. The self-obsessed teacher would irritate students with endless personal anecdotes and tales of prior adventures. The ideal teacher is the one teacher students truly respect, and internalize their advice to the point that it serves as a lesson for the rest of their life.

An effective teacher should visualize the qualities that would make him or her outstanding. This paper proposes to counter the following critiques: “*At this point, we appear to have a 19*^*th*^ *century curriculum, 20*^*th*^ *century buildings and organizations and 21*^*st*^ *century students facing an undefined future.”* (Wellman in Truss, 2010). In addition, Aromolaran (1985) critique of the lack of materials and equipment faced by the education system in many countries is a barrier to effective teaching. However, these shortcomings can be overcome by adopting Awobodu (2000) argument that teachers' utilizations of relevant equipment, materials and tools in teaching facilitates learning and enhances student achievement. Hence, in order to impart knowledge to 21st century students, an effective teacher must be able to efficiently operate basic hardware during the lesson. It should be emphasized here that a teaching aid is a tool used by teachers to help learners improve reading, writing, speaking and listening skills; illustrate or reinforce a skill, fact, or idea; and relieve anxiety, fears, or boredom.

An effective teacher should also consider factors that influence selection of teaching equipment/aids which are availability; topic of discussion; level of class/course/programme; level of complexity; students' preparedness; ease of use and suitability. For the incorporation of these aids to achieve the desired goal, there are practices that should be avoided when using any kind of teaching aids. Avoid preparing slides or posters that are too wordy, lack clarity and employ jargons; avoid reading solely from the slides; steer clear from using Power Point slides to teach quantitative subjects; do not rush and concentrate too much on particular slides only. According to Zhang et al. (2022), the learning effect of Content Based Instruction (CBI) teaching concept is more than general teaching and task-based teaching. Even the teachers' facial expression plays a crucial role in the learning process of the students. Wang (2022) investigated whether video lectures delivered by teachers with enhanced expression, or facial expression laden with a specific emotion, were more effective at promoting students' learning than lectures delivered by the teachers with conventional expression, or emotionless delivery, and the teachers' instructions via audio only. The findings indicated that for nurturing students' social presence, arousal level, and long-term learning, the video lecture by the enhanced-expression teacher was the most superior in comparison to those with the conventional-expression teachers and with the audio-only teachers. Evidently, the concept of 3H is applied in this pedagogical approach.

Although the use of technology in teaching will make the teachers appear to be relevant with the current trends of synchronous and asynchronous online learning, it does not replace the presence of the teachers. Therefore, it should be reminded that an effective teacher must always observe efficient classroom management to ensure students learning needs are met. The following are beneficial habits of an effective teacher that would help in materializing the 3H approach: implementing group discussion (mixing races and weak—good students), doing case studies, giving opportunities to students to practice in the class, using real life examples during teaching-learning sessions, summarizing lessons taught at the end of every class, giving regular feedback, using different tools and techniques and effective use of whiteboard.

## Conclusion

While teaching is a gift that comes quite naturally for some, others have to work overtime to achieve a great teacher status. Yet the payoff is enormous—for both teachers and students. Imagine students thinking of a teacher when they remember that great teacher they had in college! Teaching is a noble profession but it may turn out to be frustrating and bring negative connotations if teachers failed to perform their duty as expected of them. The unified framework of the head, heart, and hands is a flexible method to learning and teaching that may be advantageous to both teachers and students. This all-encompassing learning strategy appears to offer better opportunities for learning for education for sustainability because it engages and develops the full person, including their affective, cognitive, and practical dimensions and talents, in relation to real-world issues and concerns.

## Author contributions

Conceptualization, methodology, and supervision: MI. Original draft: SH, JU, and SA. Editing draft and supervision: FS. All authors contributed to the article and approved the submitted version.

## Funding

This article is funded by Institute for Advanced Research Publication Grant of United International University, Ref. No.: IAR-2022-Pub-035.

## Conflict of interest

The authors declare that the research was conducted in the absence of any commercial or financial relationships that could be construed as a potential conflict of interest.

## Publisher's note

All claims expressed in this article are solely those of the authors and do not necessarily represent those of their affiliated organizations, or those of the publisher, the editors and the reviewers. Any product that may be evaluated in this article, or claim that may be made by its manufacturer, is not guaranteed or endorsed by the publisher.
